# Biofilm removal from a simulated isthmus and lateral canal during syringe irrigation at various flow rates: a combined experimental and Computational Fluid Dynamics approach

**DOI:** 10.1111/iej.13420

**Published:** 2020-11-18

**Authors:** T. C. Pereira, C. Boutsioukis, R. J. B. Dijkstra, X. Petridis, M. Versluis, F. B. de Andrade, W. J. van de Meer, P. K. Sharma, L. W. M. van der Sluis, M. V. R. So

**Affiliations:** ^1^ Department of Dentistry, Endodontics and Dental Materials Bauru School of Dentistry University of São Paulo Bauru Brazil; ^2^ Center for Dentistry and Oral Hygiene University Medical Center Groningen University of Groningen Groningen The Netherlands; ^3^ Department of Endodontology Academic Centre for Dentistry Amsterdam (ACTA) University of Amsterdam and Vrije Universiteit Amsterdam Amsterdam The Netherlands; ^4^ Physics of Fluids group Technical Medical (TechMed) Center and MESA+ Institute for Nanotechnology, University of Twente Enschede The Netherlands; ^5^ Department of Orthodontics University Medical Center Groningen University of Groningen Groningen The Netherlands; ^6^ Department of Biomedical Engineering University Medical Center Groningen University of Groningen Groningen The Netherlands; ^7^ Conservative Dentistry Department School of Dentistry Federal University of Rio Grande do Sul Porto Alegre‐Rio Grande do Sul Brazil

**Keywords:** biofilm, isthmus, lateral canal, sodium hypochlorite, velocity

## Abstract

**Aim:**

(i) To quantify biofilm removal from a simulated isthmus and a lateral canal in an artificial root canal system during syringe irrigation with NaOCl at different concentrations and delivered at various flow rates (ii) to examine whether biofilm removal is further improved by a final high‐flow‐rate rinse with an inert irrigant following irrigation with NaOCl. (iii) to simulate the irrigant flow in these areas using a computer model (iv) to examine whether the irrigant velocity calculated by the computer model is correlated to biofilm removal.

**Methodology:**

Ninety‐six artificial root canals with either a simulated isthmus or lateral canal were used. A dual‐species *in vitro* biofilm was formed in these areas using a Constant Depth Film Fermenter. NaOCl at various concentrations (2, 5 and 10%) or adhesion buffer (control) was delivered for 30 s by a syringe and an open‐ended needle at 0.033, 0.083, or 0.166 mL s^−1^ or passively deposited in the main root canal (phase 1). All specimens were subsequently rinsed for 30 s with adhesion buffer at 0.166 mL s^−1^ (phase 2). The biofilm was scanned by Optical Coherence Tomography to determine the percentage of the remaining biofilm. Results were analysed by two 3‐way mixed‐design ANOVAs (α = 0.05). A Computational Fluid Dynamics model was used to simulate the irrigant flow inside the artificial root canal system.

**Results:**

The flow rate during phase 1 and additional irrigation during phase 2 had a significant effect on the percentage of the remaining biofilm in the isthmus (*P* = 0.004 and *P* < 0.001). Additional irrigation during phase 2 also affected the remaining biofilm in the lateral canal significantly (*P* ≤ 0.007) but only when preceded by irrigation at medium or high flow rate during phase 1. The effect of NaOCl concentration was not significant (*P *> 0.05). Irrigant velocity in the isthmus and lateral canal increased with increasing flow rate and it was substantially correlated to biofilm removal from those areas.

**Conclusions:**

The irrigant flow rate affected biofilm removal *in vitro* more than NaOCl concentration. Irrigant velocity predicted by the computer model corresponded with the pattern of biofilm removal from the simulated isthmus and lateral canal.

## Introduction

Root canal irrigation is one of the most important steps during root canal treatment (Gulabivala *et al*. 2005, Zehnder 2006). Its primary aim in infected root canal systems is the elimination of microorganisms that often adhere to the root canal wall and form a biofilm (Ricucci *et al*. 2009). Biofilms located in remote areas such as isthmuses, fins, oval extensions and lateral canals and also in some parts of the main root canal are beyond the reach of instruments (Peters 2004, Gulabivala *et al*. 2005, Ricucci *et al*. 2013), so irrigants are expected to disrupt and remove them by a combination of chemical and mechanical effects (van der Sluis *et al*. 2015).

The chemical effect is mainly realized by sodium hypochlorite, which remains the most widely used primary irrigant (Zehnder 2006, Dutner *et al*. 2012). Its molecules and ions are transported to the areas of interest by the bulk irrigant flow and, to a lesser extent, by diffusion (van der Sluis *et al*. 2015). Since the reactive components of NaOCl are rapidly consumed when in contact with bacteria, pulp tissue or dentine (Moorer & Wesselink 1982, Haapasalo *et al*. 2000, Portenier *et al*. 2002), frequent irrigant exchange is needed. The mechanical effect, on the other hand, is exerted via the wall shear stress applied by the flowing irrigant on the biofilm (van der Sluis *et al*. 2015). Sodium hypochlorite penetration and exchange in the main root canal and the developed wall shear stress are strongly affected by the irrigant flow rate (Boutsioukis *et al*. 2009, Verhaagen *et al*. 2012, van der Sluis *et al*. 2015) but there is very little information about the effect of the flow rate on irrigant penetration in areas beyond the main root canal, such as isthmuses and lateral canals, and on biofilm removal.

Earlier studies have also indicated that sodium hypochlorite concentration affects the disruption and removal of the biofilm following direct contact *in vitro* (Petridis *et al*. 2019b). Moreover, computer models and *in vitro* experiments have shown that sodium hypochlorite penetration into lateral canals is likely to be a diffusion‐dominated process, therefore it is expected to be directly influenced by the concentration in the main root canal (Verhaagen *et al*. 2014). However, the effect of sodium hypochlorite concentration on biofilm removal from isthmuses and lateral canals remains largely unexplored.

Whilst the chemical and mechanical effects of irrigation take place simultaneously, they are often evaluated in separate experiments (Zehnder 2006, Kishen & Haapasalo 2015, van der Sluis *et al*. 2015, Kishen *et al*. 2016). Although such simplified experiments can provide valuable data, they only allow a partial understanding of this process as the potential interactions between the two effects are ignored. In order to evaluate these effects and their interactions *in vitro,* it is necessary to mimic the geometry of the root canal system and to use a biofilm that represents the actual biofilms present in root canals *in vivo* (Swimberghe *et al*. 2019). A dense *in vitro* biofilm with realistic viscoelastic properties could indeed mimic the basal layer of the *in vivo* endodontic biofilm, which is particularly difficult to remove (He *et al*. 2013). Previous studies have established the relevance of a dual‐species biofilm (Streptococcus oralis Actinomyces naeslundii) grown in a Constant Depth Film Fermenter (CDFF) for such a purpose (Hope & Wilson 2006, Busanello *et al*. 2018, Petridis *et al*. 2019a,b) and have introduced an artificial root canal (Macedo *et al*. 2014) that can be adapted for biofilm evaluation. Due to the inevitable variability in the biofilm structure even when grown *in vitro* under strictly controlled conditions (Busscher *et al*. 2003, Busanello *et al*. 2018, Petridis *et al*. 2019a), a longitudinal evaluation method allowing measurements before and after each irrigation step, such as Optical Coherence Tomography (OCT), is indispensable for such experiments (Busanello *et al*. 2018, Swimberghe *et al*. 2019, Petridis *et al*. 2019b).

A detailed investigation of the flow developed in an isthmus and lateral canal could complement the information on biofilm removal and allow for potential correlations. Experimental high‐resolution analysis of the flow inside these areas is technically challenging even when using an artificial root canal system without any biofilm (Verhaagen *et al*. 2014). In order to circumvent these problems, a validated Computational Fluid Dynamics (CFD) model has been used in earlier studies to investigate the irrigant flow in the root canal system (Boutsioukis *et al*. 2010a,b, Verhaagen *et al*. 2014). Therefore, taking into account all the aspects discussed above, the aims of this study were:

1. To quantify biofilm removal from a simulated isthmus and lateral canal in an artificial root canal system during syringe irrigation with NaOCl at different concentrations and delivered at various flow rates and to identify any interactions between these variables.
2. To examine whether biofilm removal is further improved by a final high‐flow‐rate rinse with an inert irrigant following irrigation with NaOCl.
3. To simulate the irrigant flow in those areas using a CFD model (Boutsioukis *et al*. 2010a).
4. To examine whether the irrigant velocity calculated by the CFD model is correlated to biofilm removal in those areas.

## Materials and methods

### Sample size calculation

A sample size calculation was conducted *a priori* using G*Power 3.9 (Faul *et al*. 2007) to determine the necessary number of biofilm specimens assuming a mixed‐design ANOVA, a two‐tailed probability of alpha‐type error 0.05 and 80% power. A large effect size (*f* = 0.7) was assumed given the preliminary nature of this study, which resulted in a minimum of 3 specimens in each of the 16 groups.

### Artificial root canals and inserts

**Figure 1:**
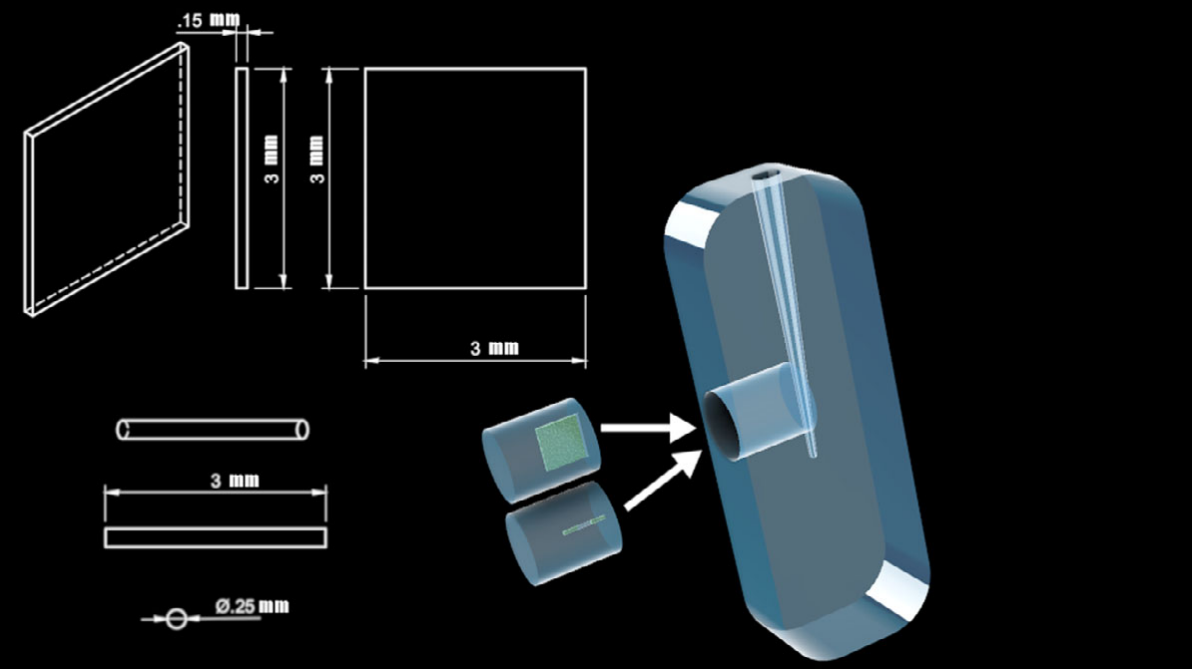
Schematic drawing of the artificial root canal and the simulated isthmus and lateral canal.

Ninety‐six transparent artificial root canals were produced by solidified Polydimethylsiloxane (PDMS) (Sylgard 184; Dow‐Corning, Midland, MI, USA) around a D‐size finger spreader (Dentsply Sirona, Ballaigues, Switzerland), as described previously (Macedo *et al*. 2014). The length of the root canals was 18 mm and the apical diameter was 0.35 mm with a taper of 6%. The moulds were modified to create a cylindrical recess (*d* = 5 mm, *l* = 6 mm) in contact with the apical third of the root canal at ~2 mm from the apical terminus. The base of the cylinder was tangent to the root canal wall (Fig. 1). The moulds were filled with degassed PDMS which was cured at 60°C for 1 h. PDMS inserts fitting the cylindrical recess were created by the same process using different moulds that included either a thin metal strip (3 × 3 × 0.15 mm, volume = 1.35 mm^3^) in order to create a simulated isthmus (*n* = 48) or a small metal cylinder (*d* = 0.25 mm, *l* = 3 mm, volume = 0.15 mm^3^) in order to create a simulated lateral canal (*n* = 48).

### Bacterial strains and growth conditions

*S. oralis* J22 and *A. naeslundii* T14V‐J1 were streaked on blood agar plates from which single colonies were used to inoculate 10 mL of modified brain heart infusion broth (37.0 g L^−1^ BHI, 1.0 g L^−1^ yeast extract, 0.02 g L^−1^ NaOH, 0.001 g L^−1^ vitamin K1, 5 mg L^−1^ L‐cysteine‐HCl, pH 7.3) (BHI, Oxoid Ltd., Basingstoke, UK), which was cultured at 37°C for 24 h in ambient air for *S. oralis* J22 and 24 hr in anaerobic conditions for *A. naeslundii* T14V‐J1 (preculture). Precultures were used to inoculate 200 mL of modified BHI, which was incubated at 37°C for 16 h (main culture). The bacteria were harvested by centrifugation (6500 × *g*) and were washed twice in sterile adhesion buffer (0.147 g L^−1^ CaCl_2_, 0.174 g L^−1^ K_2_HPO_4_, 0.136 g L^−1^ KH_2_PO_4_, 3.728 g L^−1^ KCl, pH 6.8). To break bacterial chains, bacterial suspensions were sonicated intermittently in ice water for 3 × 10 s at 30 W (Vibra cell model 375; Sonics and Materials Inc., Newtown, CT, USA). This process has been found not to cause cell lysis (van der Mei *et al*. 2008, Verkaik *et al*. 2010). The bacteria were counted in a Bürker‐Türk chamber (Marienfeld‐Superior, Lauda‐Königshofen, Germany) and diluted in sterile adhesion buffer.

### Biofilm formation

Stimulated human whole saliva was collected in accordance with the guidelines of the Medical Ethical Committee of the University Medical Centre Groningen (approval letter 06‐02‐2009). Twenty volunteers chewed Parafilm© (Sigma‐Aldrich, St Louis, MO, USA) and collected their saliva in ice‐cooled containers for 30 min. The collected saliva was pooled, centrifuged, stabilized by adding a protease blocker and freeze‐dried. Reconstituted human Whole Saliva (RWS) was obtained by dissolving freeze‐dried saliva in adhesion buffer at a concentration of 1.5 g L^−1^, stirring for 2 h and centrifuging at 10 000 × *g* at 10°C for 5 min. The PDMS inserts with the simulated isthmus or lateral canal were coated with RWS through static exposure for 14 h at 4°C.

A CDFF was used to grow steady‐state dense cell‐rich biofilms with standardized structural properties, similarly to previous studies (Busanello *et al*. 2018, Petridis *et al*. 2019a,b). The CDFF consisted of a rotating turntable which held 15 sample holders with 5 sample spots each. Biofilm could be formed in these holders under mechanical compaction by fixed scraper blades. All components of the CDFF were initially immersed in 0.2% Peracetic acid for 24 h and the CDFF along with the inserts and culture media were autoclaved for 30 min at 121 ºC in order to prevent contamination. The saliva‐coated inserts were transferred to the sample holders on the turntable. Dropwise inoculation of 100 mL of the dual‐species bacterial suspension, containing *S. oralis* J22 (6 × 10^8^ bacteria mL^−1^) and *A. naeslundii* T14V‐J1 (2 × 10^8^ bacteria mL^−1^), took place in the CDFF over 1 h whilst the turntable rotated slowly at a constant speed (3 rpm). Next, rotation was stopped and the bacteria were allowed to adhere for 30 min to the saliva‐coated inserts. Rotation was then resumed and modified BHI was continuously supplied (45 mL h^−1^) so that biofilms could develop during the next 96 h at 37°C. The fixed scraper blades applied the necessary pressure and distributed nutrients over the inserts for biofilm development.

Subsequently, the biofilm‐filled inserts were removed from the CDFF and they were placed inside a jar containing adhesion buffer to prevent dehydration. Immediately before use they were removed from the jar and fitted in the cylindrical recess of the artificial root canal creating a fluid‐tight apically sealed system. The biofilm was scanned by OCT prior to the irrigation experiments.

### Irrigation experiments

Three different NaOCl solutions (2, 5 and 10%) were freshly prepared from a 12–15% stock solution (Sigma‐Aldrich). Their concentration was verified by iodometric titration. The adhesion buffer was used as a negative control (0% NaOCl). The PDMS inserts were randomly allocated to 4 groups with a simulated isthmus (*n* = 12) and another 4 groups with a simulated lateral canal (*n* = 12) according to the irrigant flow rate. Each of these groups was further divided into 4 subgroups according to the NaOCl concentration used (*n* = 3). Irrigation was carried out in two phases:

During phase 1 the specimens in each group were irrigated by a 5‐mL syringe (Ultradent Products Inc, South Jordan, UT, USA) and a 30G open‐ended needle (Navitip; Ultradent Products Inc) at a steady flow rate (0.033, 0.083 or 0.166 mL s^−1^) for 30 s or the irrigant was passively deposited in the root canal until it was completely filled and then left undisturbed for 30 s in order to mimic a purely chemical effect (flow rate = 0 mL s^−1^). The needle was initially placed at 2 mm from the apical end‐point of the root canal and it was also moved along the root canal during irrigation (between 1–5 mm). Within each group, the specimens were irrigated with either 2, 5 or 10% NaOCl or adhesion buffer, according to the subgroup allocation. Sodium thiosulfate 4.23% (Sigma‐Aldrich) was subsequently deposited passively in all the root canals to neutralize any remaining NaOCl and the biofilm was scanned again by OCT.

Phase 2 consisted of a final 30‐second rinse with adhesion buffer delivered at 0.166 mL s^−1^ for all subgroups using the same syringe irrigation protocol as in phase 1. Afterwards, the biofilm was scanned by OCT for a third time.

### Optical coherence tomography and image analysis

The biofilm in the simulated isthmus and lateral canal was scanned by an OCT scanner (Thorlabs, Newton, NJ, USA) three times (before irrigation, after phase 1 and after phase 2) using a 45‐mm field of view and a refraction index of 1.33 in order to obtain real‐time three‐dimensional renderings of the remaining biofilm. The scans were initially processed with ThorImage OCT software (Thorlabs) and pre‐ and postirrigation scans were exported to the open‐source image analysis software Fiji 1.50g (Schindelin *et al*. 2012) . The scans were processed in order to segment biofilm from voids and to calculate the volume of the isthmus or lateral canal that was occupied by biofilm. The biofilm volumes after phase 1 and phase 2 were divided with the biofilm volume before irrigation to calculate the percentage of remaining biofilm in the isthmus or lateral canal after each irrigation phase.

### Statistical analysis of biofilm removal

The effect of the flow rate and NaOCl concentration during phase 1 and of the additional irrigation during phase 2 were analysed separately for the simulated isthmuses and lateral canals by two 3‐way mixed‐design ANOVAs. The percentage of the remaining biofilm was selected as the dependent variable. Normality was verified by the Shapiro–Wilk test and equality of error variances was assessed by Levene’s test. The null hypotheses were that the flow rate and the NaOCl concentration during phase 1, and the additional irrigation during phase 2 have no significant effect on the amount of remaining biofilm. Tukey’s honestly significant difference post hoc test was employed for pair‐wise comparisons. The alpha level was set to 0.05. Bonferroni correction for multiple comparisons was applied to this level where appropriate. Confidence intervals (95% CI) of the differences between groups were also calculated. Statistical analysis was performed using SPSS 23 (IBM Corp, Armonk, NY).

### Computational Fluid Dynamics model

The irrigant flow inside the artificial root canal system without any biofilm was studied using a previously validated CFD model (Boutsioukis *et al*. 2010a,b, Verhaagen *et al*. 2014). The irrigant was delivered by a 30G open‐ended needle (NaviTip; Ultradent) which was modelled as described previously (Boutsioukis *et al*. 2010b). Since the needle was moved along the root canal during the experiments, five different cases were simulated (needle tip at 1, 2, 3, 4 and 5 mm from the apical end‐point) using the highest irrigant flow rate (0.166 mL s^−1^) as a preliminary step to determine the optimum needle position regarding irrigant penetration into the isthmus and lateral canal. Additional cases were then simulated with the needle at the optimum position using the other two flow rates (0.083 and 0.033 mL s^−1^). A hybrid mesh (1.4–4.3 million cells) was constructed and refined near the walls and in the isthmus/lateral canal. Grid‐independence of the results was also verified. Sodium hypochlorite 5% [density = 1.09 g cm^−3^ and viscosity = 1.11·10^−3^ Pa·s (Guerisoli *et al*. 1998)] was modelled as the irrigant in all cases. The commercial CFD solver Fluent 14.5 (ANSYS Inc., Canonsburg, PA, USA) was used to set up and solve the problem (Boutsioukis *et al*. 2010a). Computations were carried out in a workstation with a 6‐core Intel Xeon 3.2 GHz processor (Intel, Santa Clara, CA, USA) and 32 GB of RAM. The flow fields for the 6 simulated cases with the needle at the optimum position were compared in terms of time‐averaged irrigant velocity and wall shear stress.

### Correlation between biofilm removal and irrigant velocity

The percentage of the biofilm removed along a narrow plane in the middle of the simulated isthmus (thickness = 3 voxels) and lateral canal (thickness = 1 voxel) was calculated separately for each specimen based on the OCT scans. The results from the different subgroups were combined in order to calculate the average percentage of biofilm removed for each flow rate since irrigant velocities calculated by the CFD model were independent of the NaOCl concentration.

The spatial correlation between the log‐transformed irrigant velocity calculated by the CFD model and the average percentage of biofilm removed from the simulated isthmuses and lateral canals was examined using Pearson’s correlation coefficient. The log‐transformation was used because the velocity inside the isthmus and lateral canal spanned several orders of magnitude and its distribution was highly skewed. In order to increase the signal‐to‐noise ratio of the OCT scans and to account for minor experimental misalignments, an interrogation window of 10 × 10 pixels was chosen; CFD data were downsampled accordingly. Data from the three different flow‐rate groups (0.033, 0.083 and 0.166 mL s^−1^) were pooled to increase the sample size. Simple linear regression models were also fitted to the data.

## Results

### Remaining biofilm

**Figure 2:**
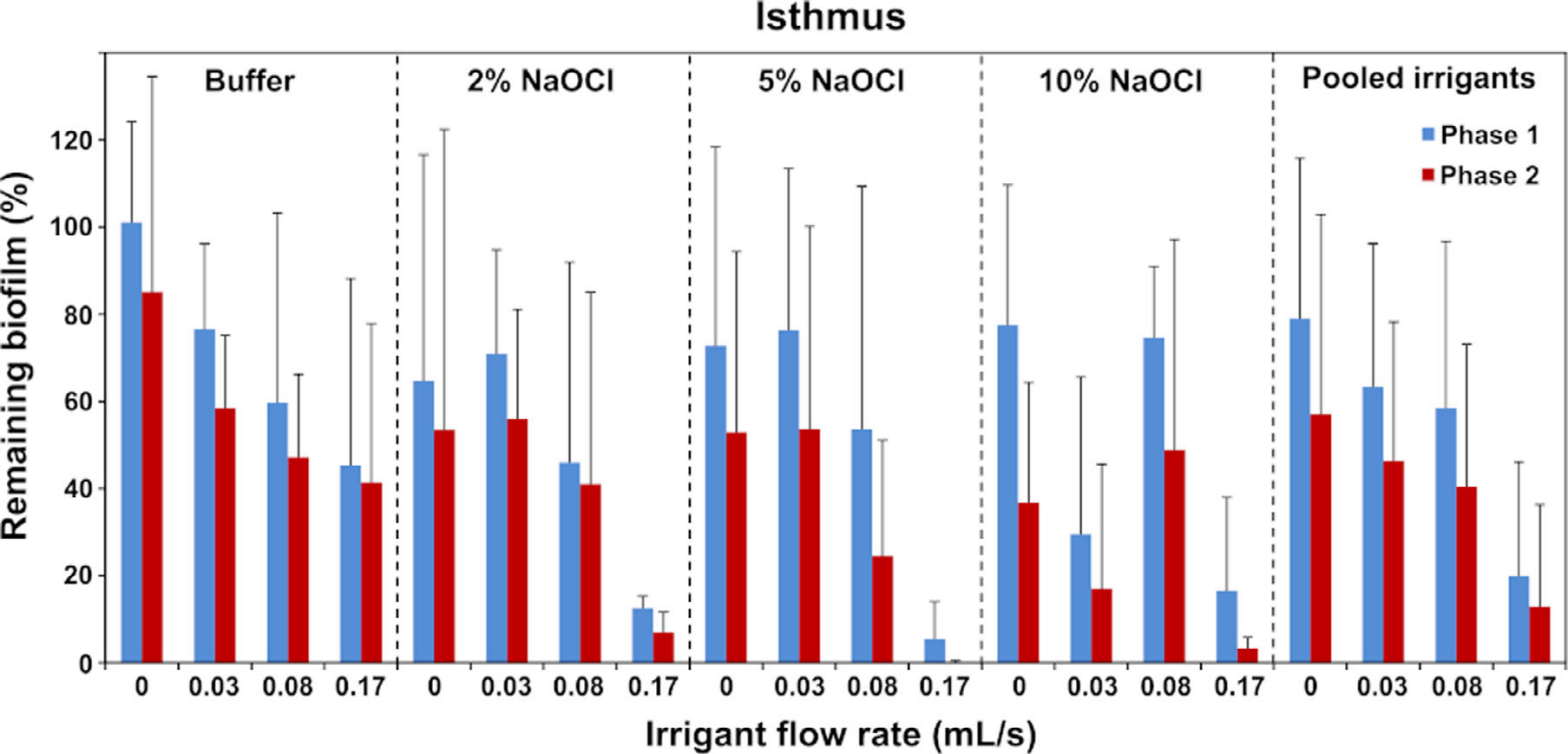
Biofilm remaining in the simulated isthmus after phase 1 and phase 2 expressed as average percentage of the initial biofilm volume, for the various irrigants and flow rates. Error bars indicate standard deviations.

**Figure 3:**
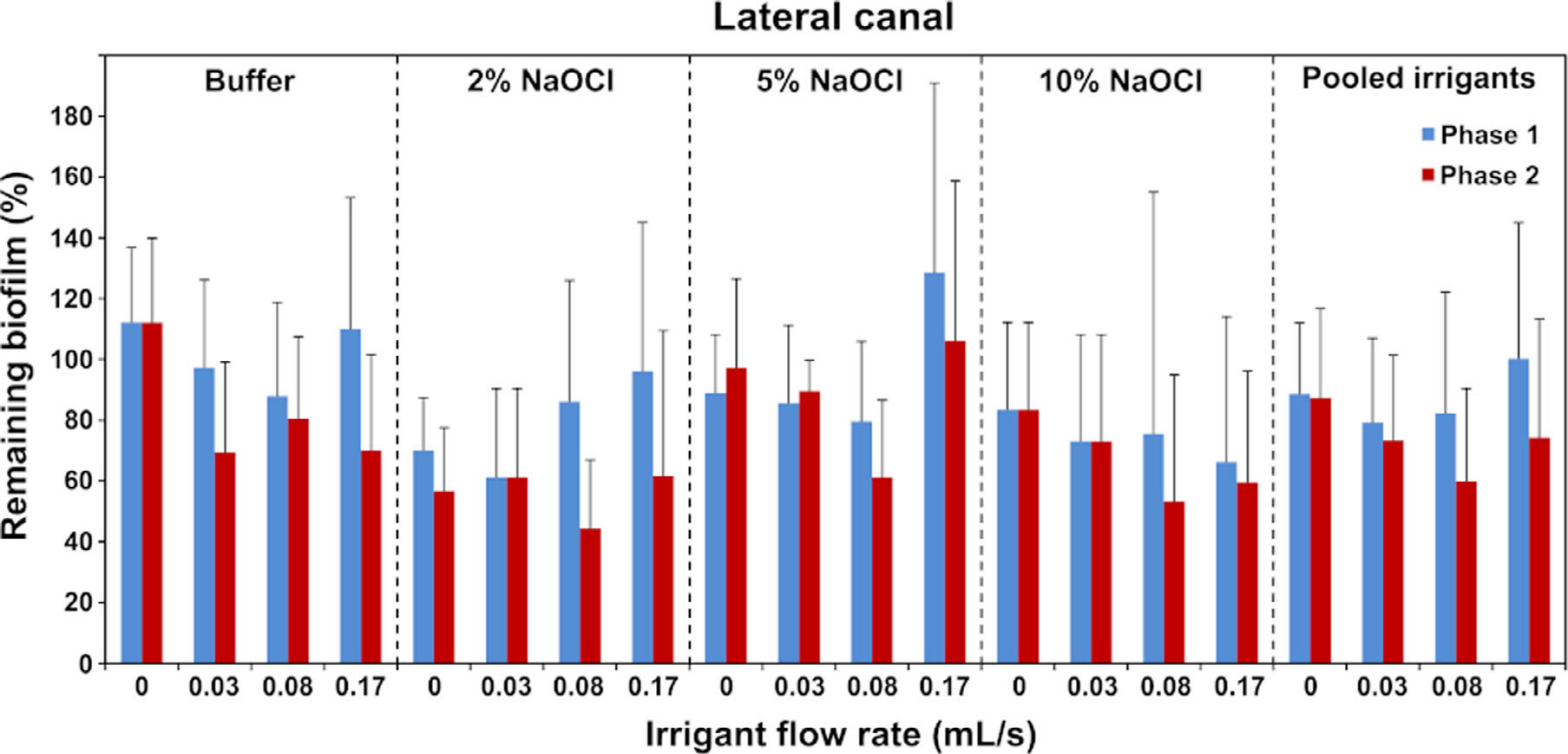
Biofilm remaining in the simulated lateral canal after phase 1 and phase 2 expressed as average percentage of the initial biofilm volume, for the various irrigants and flow rates. Error bars indicate standard deviations.

The mean initial biofilm volume in the simulated isthmus was 0.809 mm^3^ (SD 0.339), so approximately 60% of the isthmus was occupied. The mean initial biofilm volume in the simulated lateral canal was 0.025 mm^3^ (SD 0.009) (approximately 17% of the total lateral canal volume); the biofilm was organized in plugs (dense masses of biofilm) near the entrance and the distal part. Irrigation generally resulted in a decrease in the biofilm volume after both phase 1 and phase 2 without achieving total elimination but an increase was also observed in some groups (Figs 2 and 3).

Regarding the isthmus, none of the interactions between the independent variables were significant, so the main effects were interpreted. The flow rate during phase 1 had a significant effect on the percentage of the remaining biofilm (*P* = 0.004). *Post hoc* comparisons showed that a flow rate of 0.166 mL s^−1^ resulted in significantly less biofilm than 0 and 0.033 mL s^−1^ (*P* = 0.030; 95% CI: 15.1–88.0% and *P* = 0.036; 95% CI: 1.9–74.8%, respectively). No other significant differences were found amongst the various flow rates. Additional irrigation during phase 2 also reduced the amount of biofilm significantly, irrespective of other parameters (*P* < 0.001; 95% CI: 8.5–23.5%). The effect of the NaOCl concentration was not significant (*P* = 0.229).

In the lateral canal, the main effect of the NaOCl concentration was not significant (*P* = 0.131) but the interaction between the phase and the flow rate was significant (*P* = 0.005), so a simple‐effects analysis took place for these two variables. The effect of the irrigation during phase 2 was significant only after irrigation at 0.083 or 0.166 mL s^−1^ during phase 1 (*P* = 0.007; 95% CI: 7.5–37.4% and *P* = 0.001; 95% CI: 12.8–39.1%), irrespective of the NaOCl concentration. The effect of irrigant flow rate was not significant for any of the two phases (*P* = 0.478 and *P* = 0.233). None of the other interactions between the independent variables were significant (*P > 0.1*).

### Irrigant flow

**Figure 4:**
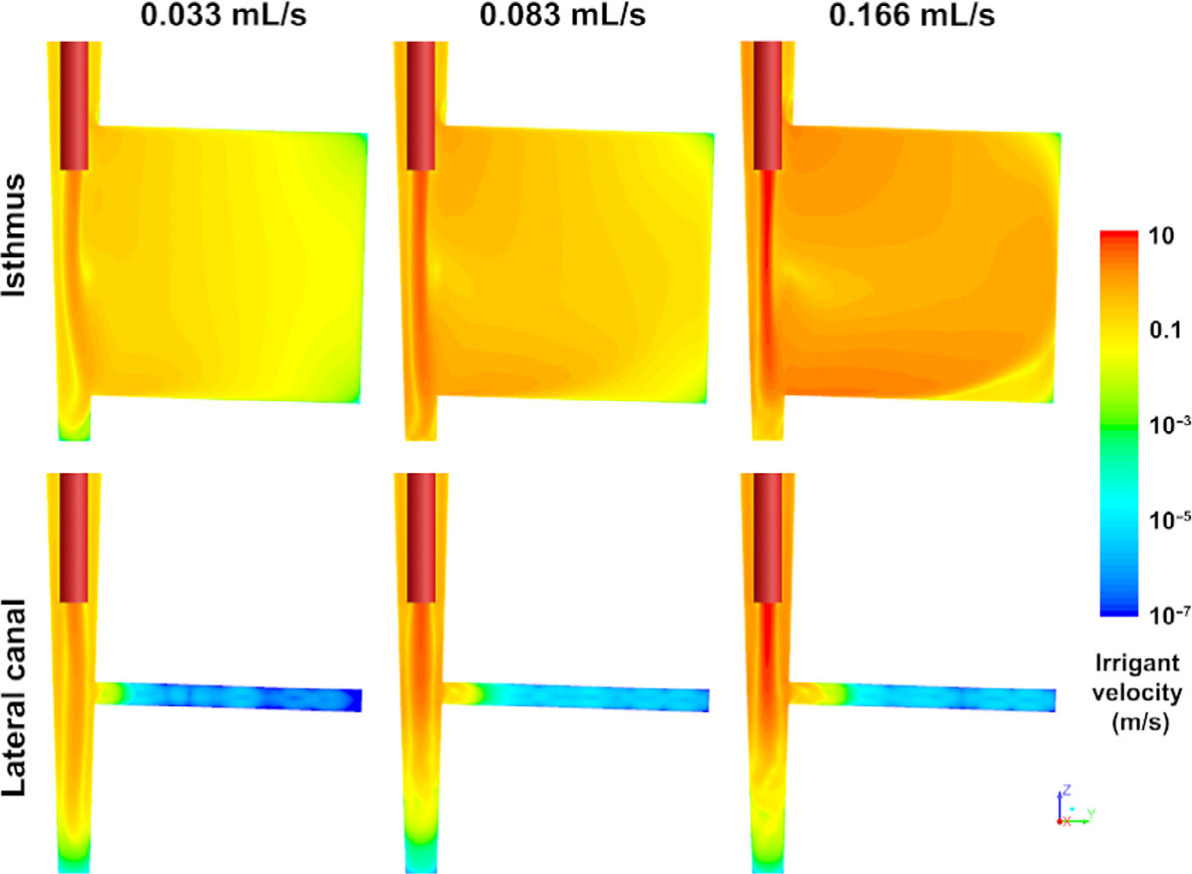
Contours of time‐averaged irrigant velocity magnitude along the middle plane in the apical part of the main root canal and the isthmus/lateral canal for the three different irrigant flow rates simulated by the CFD model. A logarithmic scale has been used. The open‐ended needle (in red) was inserted at 3 mm from WL. Extremely low irrigant velocities (<10^−6^ m s^−1^) were calculated in some areas of the lateral canal, which could indicate irrigant stagnation in those areas.

The optimum position of the needle was at 3 mm from the apical end‐point of the root canal concerning both the isthmus and the lateral canal. This position led to the highest time‐averaged velocities (Fig. 4). A qualitative comparison between the various simulated cases showed that the irrigant velocity and wall shear stress in these areas increased with increasing irrigant flow rate. Extremely low irrigant velocities (<10^−6^ m s^−1^) were calculated in some parts of the lateral canal, which could indicate irrigant stagnation. A comparison of the CFD results to the biofilm removal observed during the experiments showed that the irrigant velocity was higher in areas where the biofilm was removed more effectively.

### Correlation between irrigant velocity and biofilm removal

**Figure 5:**
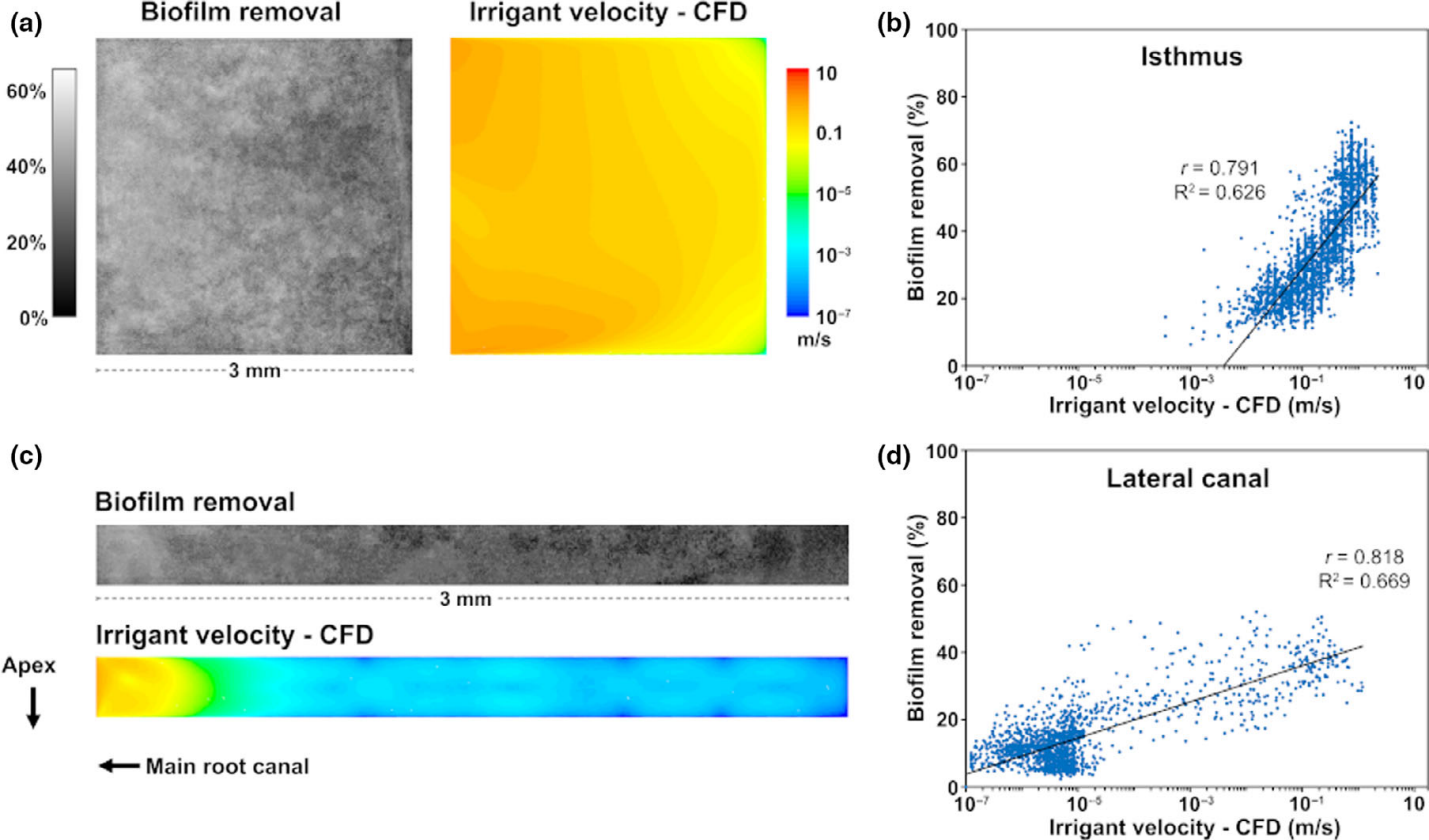
Average percentage of biofilm removed from the middle of the simulated isthmus (a) and lateral canal (c) during syringe irrigation at 0.083 mL s^−1^for all irrigants tested (grayscale images), contours of time‐averaged irrigant velocity calculated by the CFD model in the same area (coloured images), and corresponding scatter plots of the pooled data from all three flow rates indicating the correlation of the two variables (b and d). Each dot represents an area of 10 × 10 pixels inside the isthmus or lateral canal. Simple linear regression models have been fitted to the data.

There was a substantial spatial correlation between the log‐transformed irrigant velocity calculated by the CFD model in the isthmus and lateral canal and the percentage of biofilm removed from these areas (*r* = 0.79 and *r* = 0.82, respectively; Fig. 5). The linear regression models indicated that 62.6% and 66.9% of the total variance in the biofilm removal in these areas could be explained by the irrigant velocity. Furthermore, there was a threshold velocity in the isthmus (~0.004 m s^−1^) below which no biofilm removal was expected. No such threshold could be identified for the lateral canal.

## Discussion

This study tested the hypothesis that the flow rate and NaOCl concentration may influence the removal of biofilm from isthmuses and lateral canals. The CDFF biofilm used is an *in vitro* biofilm replicating dental plaque, particularly regarding its physical properties. The densely formed biofilm with strong interactions between the microorganisms resembles the basal layer of root canal biofilm (Hope & Wilson 2006, Derlon *et al*. 2008, Ricucci & Siqueira 2010) and especially cell‐rich biofilm formed under space limitations in isthmuses or lateral canals (Ricucci & Siqueira 2010, Busanello *et al*. 2018). Both of these types of biofilm are very challenging to remove (Ricucci & Siqueira 2010, He *et al*. 2013).

Artificial root canal systems made of a transparent material (PDMS) were used instead of extracted teeth to allow standardization of the geometry (Macedo *et al*. 2014) and repeated evaluation of the biofilm by OCT. Biofilm can adhere to PDMS (Song & Ren 2014, Layton *et al*. 2015, Song *et al*. 2015) and the dual‐species biofilm of *S. oralis* and *A. naeslundii* produces a strong internal network which improves biofilm cohesion and adhesion on saliva‐coated surfaces (He *et al*. 2013, Song *et al*. 2015, Busanello *et al*. 2018). Nevertheless, in the absence of reliable *in vivo* evidence it remains unclear whether the adhesion of the *in vitro* biofilm to PDMS is similar to the adhesion of real endodontic biofilm on dentine *in vivo*.

An open‐ended needle was used for irrigant delivery during the experiments and computer simulations because the unsteady jet created by this needle can penetrate farther in the main root canal and irrigant exchange is more effective compared to a closed‐ended needle (Boutsioukis *et al*. 2010b). Moreover, its axisymmetric shape eliminated the confounding effect of the vent orientation in relation to the isthmus and lateral canal entrance, which could have been a problem with a closed‐ended needle.

The main outcome measure was the remaining biofilm volume which was determined before and after each experiment in a nonintrusive way by OCT. This longitudinal evaluation method provided information about the complete simulated isthmuses and lateral canals and allowed for each sample to be used as its own control, so individual variations in the biofilm structure and volume could be accounted for (Busanello *et al*. 2018). However, OCT cannot ascertain the condition of the bacteria (intact or damaged) in the remaining biofilm. Other methods such as Confocal Laser Scanning Microscopy (CLSM) in combination with fluorescent staining could have provided such information (Swimberghe *et al*. 2019) but at the expense of the repeated measurements. OCT has been used in parallel with CLSM in previous studies that demonstrated its validity (Busanello *et al*. 2018, Petridis *et al*. 2019a,b) and showed that the remaining cell‐rich biofilm still contained viable microorganisms (Busanello *et al*. 2018). It is noteworthy that a recent review recommended using biofilm removal as an outcome measure instead of bacterial killing because of the various bacterial components that can still induce apical pathosis even after bacteria are killed (Swimberghe *et al*. 2019).

One of the aims of this study was to investigate potential interactions between the chemical and mechanical effects of irrigation, using the NaOCl concentration and the flow rate as proxies. Phase 1 was designed to resemble a final irrigation with NaOCl after instrumentation. Even though a 30‐second rinse may appear too short, isthmuses and lateral canals are unlikely to be reached by irrigants before instrumentation is completed *in vivo*. No EDTA or other chelators were used in order to reduce the number of variables and to isolate the effect of NaOCl. Phase 2 was added to evaluate the potential mechanical cleaning effect of an additional rinse with an inert irrigant (no chemical effect) delivered at high flow rate after pretreatment of the biofilm with various combinations of chemical and mechanical effects during phase 1. Such a rinse with an inert irrigant would probably carry a very low risk of tissue damage in case of inadvertent extrusion through the apical foramen. However, it is not part of current clinical protocols; chemically active irrigants are used instead.

The experiments demonstrated the significant effect of flow rate during irrigation in phase 1 and the additional effect of a high‐flow‐rate final irrigation with an inert solution (buffer) regarding the isthmus. Therefore, a strong mechanical effect appeared to be important for the removal of the biofilm during both phases. In the lateral canal, only the final rinse with the buffer had a significant effect on biofilm removal and this effect appeared only when preceded by irrigation at medium or high flow rate during phase 1. Thus, there was a synergistic effect between a strong mechanical effect in phase 1 and the additional mechanical effect in phase 2.

NaOCl concentration did not affect the results significantly in the simulated isthmus and lateral canal, which appears to be at variance with earlier studies (Macedo *et al*. 2010, Verhaagen *et al*. 2014, Petridis *et al*. 2019b). NaOCl is not consumed by PDMS, as is the case with dentine, therefore all the free available chlorine could react with the biofilm. Irrigant penetration in lateral canals is dominated by diffusion except for a small area near their entrance where a convective flow can develop. Thus, the concentration of the irrigant in the main root canal should be directly related to its effect in the rest of the lateral canal. (Verhaagen *et al*. 2014). However, the shorter application time in this study (30 instead of 600 s), which hampered diffusion, could be an explanation for this difference. Another possible explanation is that the biofilm created in this study was mainly located near the entrance, an area that could be reached easily by the flow, and near the distal end, an area unlikely to be reached by either the flow or by diffusion within the time‐frame of the experiment. The middle part of the lateral canal, where diffusion would dominate, was almost empty. In addition, only the remaining biofilm volume was evaluated and not the viability of the bacteria within that volume, which could have been affected by the concentration of NaOCl. Moreover, it should be emphasized, regarding both isthmuses and lateral canals, that this study aimed to detect only relatively large effects and the sample size was selected accordingly. NaOCl concentration may still have some effect on biofilm removal from isthmuses and lateral canals, although this effect is likely to be smaller than that of the irrigant flow rate.

Despite irrigation with NaOCl, which is a strong antimicrobial solution, an increase was observed in the biofilm volume in some of the groups. Similar sporadic findings have also been reported in earlier studies and they have been attributed to the reaction of the biofilm to chemical and mechanical stress (Busscher *et al*. 2003, Busanello *et al*. 2018, Petridis *et al*. 2019a). Volumetric biofilm expansion may also explain some of the variance within each subgroup and it would have remained undetected if the biofilm had been evaluated only after irrigation. This highlights the importance of the repeated measurements allowed by OCT.

A substantial correlation was found between irrigant velocity in the isthmus and lateral canal and biofilm removal, a finding that validates the use of irrigant velocity as a predictor for biofilm removal. Still, only around 65% of the variance in biofilm removal could be explained by the velocity. Biofilm disruption and removal could also be mediated by the chemical effect of NaOCl to some extent but chemical interactions were not included in the computer model. Furthermore, the model assumed that the isthmus and lateral canal were free of biofilm because the detailed physical interaction between the irrigant and the biofilm exceeds current computer modelling capabilities. Therefore, the calculated velocity was a good approximation of the actual velocity in relatively clean isthmuses/lateral canals but it deviated from the actual velocity when large amounts of biofilms were present.

## Conclusions

The NaOCl flow rate during phase 1 and additional irrigation with an inert irrigant at high flow rate during phase 2 had a significant effect on *in vitro* biofilm removal from the simulated isthmus. Additional irrigation during phase 2 also affected biofilm removal from the simulated lateral canal significantly but only when preceded by irrigation at medium or high flow rate during phase 1. NaOCl concentration did not affect biofilm removal from this artificial root canal system significantly. Total elimination of the *in vitro* biofilm was not achieved consistently in any group. Higher irrigant velocity and wall shear stress were calculated in the isthmus and lateral canal by the computer model when the flow rate was increased. The irrigant velocity was substantially correlated to *in vitro* biofilm removal from those areas.

## Conflict of interest

Dr. Thais Pereira reports grants from Coordenação de Aperfeiçoamento de Pessoal de Nível Superior ‐ CAPES and Abel Tasman Talent Program. Dr van der Sluis report a grant from the European Society of Endodontology. Dr Marcus So reported a grant from CNPq. The other authors have stated explicitly that there are no conflicts of interest in connection with this article.
